# Associations of reading language preference with muscle strength and physical performance: Findings from the Integrated Women’s Health Programme (IWHP)

**DOI:** 10.1371/journal.pone.0284281

**Published:** 2023-04-10

**Authors:** Joelle Tan Hwee Inn, Beverly W. X. Wong, Yiong Huak Chan, Huang Zhongwei, Susan J. S. Logan, Jane A. Cauley, Michael S. Kramer, Eu-Leong Yong

**Affiliations:** 1 Yong Loo Lin School of Medicine, National University of Singapore, Singapore, Singapore; 2 Department of Obstetrics and Gynaecology, National University Hospital, Yong Loo Lin School of Medicine, National University of Singapore, Singapore, Singapore; 3 Biostatistics Unit, Yong Loo Lin School of Medicine, Singapore, Singapore; 4 NUS Bia-Echo Asia Centre for Reproductive Longevity and Equality, Yong Loo Lin School of Medicine, Singapore, Singapore; 5 Institute of Molecular and Cell Biology, Agency of Science, Technology and Research, Singapore, Singapore; 6 Department of Epidemiology, University of Pittsburgh, School of Public Health, Pittsburgh, PA, United States of America; 7 Faculty of Medicine, Departments of Epidemiology, Biostatistics & Occupational Health and of Pediatrics, McGill University, Montreal, Quebec, Canada; National Healthcare Group, SINGAPORE

## Abstract

**Background:**

The contribution of language preference and ethnicity to muscle strength and physical performance is unclear. We examined the associations of reading language preferences with muscle strength and performance in Chinese women and compared them to other ethnicities.

**Methods:**

The Integrated Women’s Health Programme (IWHP) cohort comprised community-dwelling, midlife Singaporean women aged 45–69. Ethnic Chinese women could choose between the English or Chinese versions of the questionnaire. Malay and Indian women were presented with the English version. Sociodemographic, reproductive, anthropometric characteristics were obtained. Hand grip strength and physical performance were objectively assessed. Visceral adiposity (VAT) was determined by Dual-energy X-ray Absorptiometry. Multivariable logistic regression models were used to determine independent associations of language preference/ethnicity with muscle strength and physical performance.

**Results:**

The cohort comprised 1164 women (mean age: 56.3±6.2 years); 84.1% Chinese, 5.6% Malay, and 10.3% Indian. 315 Chinese participants (32.2%) had a Chinese-language reading preference (CLP). CLP women tended to be parous, of a lower socioeconomic status (lower proportions received tertiary education, lower employment rates and lower household income), and engaged in less physical activity compared to Chinese women with an English-language preference (ELP). This translated to a weaker hand grip strength (aOR: 1.56; 95%CI: 1.07–2.27), slower repeated chair stand (1.55; 1.12–2.13), poorer balance on tandem stand (2.00; 1.16–3.47), and a slower gait speed (1.62; 1.06–2.47). Compared to ELP women, Malay women had higher odds of poor hand grip strength (1.81; 1.12–2.93) while Indians had a higher odd of poor balance on one-leg stand (2.12; 1.28–3.52) and slow gait speeds on usual (1.88; 1.09–3.25) and narrow walks (1.91; 1.15–3.17).

**Conclusions:**

Chinese language reading preference was associated with inferior muscle strength and physical performance. Such disparities were largest and most consistent in the CLP group, followed by Indian and Malay women compared to the ELP group. Further studies should determine if CLP-associated muscle weakness can predict adverse health outcomes.

## 1. Introduction

Ageing results in a pronounced decline in muscle strength and performance commencing around the fourth decade of life [1, 2]. Muscle strength, but not muscle mass, has been associated with mortality in the Health, Aging and Body Composition (Health ABC) cohort, with declines in muscle strength leading to increased frailty, falls, immobility, and inability to perform activities of daily living [1–3]. Weak handgrip strength has been associated with increased all-cause mortality and higher death rates from cardiovascular diseases, respiratory diseases, and cancers [4–6]. Measures of lower body strength and function, such as the repeated chair stand test and timed walk tests, have also been associated with mortality [7, 8]. Moreover, the age-related decline in muscle strength carries an enormous economic burden, with the cost of hospitalization in patients with sarcopenia estimated at $40.4 billion [9, 10]. It is therefore important to identify factors that adversely affect muscle strength [9].

Ethnicity has been associated with physical performance, with poorer functional status reported among Blacks and Hispanics than among non-Hispanic White populations [11]. In a mediation analysis from the US Study of Women’s Health Across the Nation (SWAN), disparities in physical performance between White and Chinese women were attributed directly to ethnicity after accounting for confounders such as socioeconomic status, education, comorbidities, body mass index (BMI), and physical activity [12, 13]. However, Sternfeld *et al*. acknowledged that their study may have failed to capture other factors embedded within the sociocultural and behavioural context [12, 14, 15]. Language preference, possibly a reflection of an individual’s cultural beliefs, values, and social network [16], has been established as a determinant of health-seeking behaviours [17, 18]. Given the interplay between culture and language, language preferences may help understand racial-ethnic disparities in muscle function [19].

Independent of ethnicity, non-English language preference in an English-dominant environment has been established as a risk factor for cardiovascular diseases [20]. While language preference has been studied extensively as a social determinant of health, it is usually studied in the context of healthcare access [21]. Few studies have explored the association of language preference in relation to muscle strength and performance in women from a single ethnicity [22]. Even fewer have examined disparities in physical performance among Asians; most have been based on Western populations [23]. Those few studies have aggregated Asian-American subgroups living in western educational and cultural environments, which may mask biological and sociocultural differences among Asian subgroups and lead to incorrect interpretations [24]. To the best of our knowledge, no study has examined the association between language preference and objectively measured physical performance across various ethnicities in an Asian population. Addressing this knowledge gap is particularly important for midlife women, who experience an accelerated decline in muscle strength at about the fifth decade of life [25].

Our purpose was to determine if reading language preference (English or Chinese) among the midlife ethnic Chinese women is associated with differences in muscle strength and performance. We also assessed differences in objectively assessed physical performance indices comparing ethnic Chinese who prefer to use English with Malay and Indian women. Since considerable differences in lifestyles have been reported to be associated with language preference, and racial/ethnic disparities have been found to be associated with physical performance in midlife Women in the USA, we hypothesised that differences in muscle function and performance would be observed both within and between ethnicities [12, 26].

## 2. Methodology

### 2.1. Study population

The Integrated Women’s Health Programme (IWHP) is a prospectively enrolled cohort of community-dwelling Singaporean women aged betweenl 45 and 69, conceptualised to identify the healthcare needs of midlife Asian women. The cohort was enrolled between 2014 to 2016. Besides completing a series of validated questionnaires, we also obtained objective biophysical measurements such as muscle strength and physical performance. Women were excluded if they did not possess the minimal level of literacy to independently complete the questionnaire and provide informed consent. This was defined as the inability to read at least three words (n = 46) on the Rapid Estimate of Adult Literacy in Medicine Short Form (REALM-SF), a validated tool to measure literacy levels [27]. Of the 2191 women eligible for inclusion, 990 women declined or were non-contactable, leaving us with a cohort of 1201 women who completed the baseline visit. Information related to ethnicity was provided by 58.0% of women who declined. The proportion of participants who enrolled compared to those who declined did not differ significantly between the Chinese and Malay ethnicities. Notably, a significantly greater proportion of Indian women consented to enrolment compared to the two other ethnic groups. All women provided informed consent, and the study was approved by the National Healthcare Group (NHG) Domain Specific Review Board (DSRB) (reference number 2014/00356). Details of the cohort have been described elsewhere [28].

### 2.2. Key exposure variables: Ethnicity and language preference

Ethnic categorisation was based on the participant’s self-reported ethnicity. Participants were requested to choose from the following options: Chinese, Malay, Indian, Eurasian, Others, Don’t Know, and Refused. Reading language preference was established by the language in which the baseline questionnaire administered (English or Chinese). The questionnaire was available only in English or Chinese for the following reasons. First, Chinese are the ethnic majority in Singapore at 74.3%; and among the Chinese, the 2015 General Household Survey revealed that 19.4% of ethnic Chinese were exclusively literate in their mother tongue, compared to only 9.9% of Malays and 3.6% of Indians [29]. Second, the number of Malay and Indian participants recruited in our study was relatively small, and hence we did not offer versions of the questionnaire in Malay, Tamil, or other Indian languages. In other words, the English version of the questionnaire was administered to all Malay and Indian participants. The main ethnicity/language exposure variable was therefore grouped into four categories: (1) Chinese-language preference (CLP) Chinese, (2) English-language preference (ELP) Chinese, (3) Malay, and (4) Indian.

### 2.3. Primary outcome measures

Muscle strength and physical performance were assessed using standardised protocols by trained personnel [28]. Handgrip strength (kg) was measured using a hand dynamometer (Jamar, Bolingbrook, IL) as per methods set out by the American Society of Hand Therapists [30]. The maximum hand grip strength over four readings, two for each hand, was then dichotomised as <18 kg and or ≥18 kg, as recommended by the Asian Working Group for Sarcopenia (AWGS) [31]. The Short Physical Performance Battery (SPPB) was modified using the protocol described by Simonsick *et al*. [32]. We adopted this validated method to discriminate gradations of higher-level functioning in our cohort of healthy, community-dwelling midlife women. This enhanced battery of tests has three timed components: (1) five repeated chair stands (not involving arms) as a measure of lower limb strength, (2) static standing balance test in three positions for 30 seconds each (semi-tandem stand, tandem stand, and one-leg stand), and (3) 6-m walk (usual and narrow to assess functional mobility and balance, respectively) [32]. Previously established cut-offs were used to dichotomize the performance. Poor performance for each of these outcomes was defined as ≥12 seconds for the five-repetition chair stand test; <30 seconds for the semi-tandem stand tandem stand and one-leg stand; and <1m/s for the usual gait speed and narrow gait speed [33, 34]. The Muscle Strength Index (MSI), first described by Wong *et al*., is a composite measure assessing combined upper and lower body muscle strength [35]. Cut-off values for upper extremity strength, quantified by hand grip strength, and for lower extremity strength, quantified by time taken to complete five repeated chair stands, were adopted from the AWGS [31]. The classification is as follows: poor overall strength (hand grip strength <18 kg *and* five repeated chair stands ≥12 seconds), intermediate (hand grip strength <18 kg *or* five repeated chair stands ≥12 seconds), or normal (hand grip strength 18 kg *and* five repeated chair stands <12 seconds) [35].

### 2.4. Key covariates

#### 2.4.1. Baseline questionnaire

We adapted a comprehensive self-administered questionnaire from the Mobility and Independent Living in Elders Study (MILES) [28]. It features 281 questions addressing sociodemographic features, reproductive health and status, lifestyle habits, and self-rated health at baseline. Validated versions of the forms in Chinese were used when available, and when unavailable, we used an established translation procedure involving forward and backward translation. Sociodemographic features of interest included age, marital status (married/not married), highest educational attainment (no formal education or primary school/secondary school or pre-tertiary/tertiary), employment status (working/not working), and monthly household income (<SGD 3000/3000-6999/7000 and above). Reproductive health and status characteristics included parity (nulliparous/multiparous), menopausal status (premenopausal/perimenopausal/postmenopausal), and age at menopause [28]. Menopausal status was defined as follows: premenopausal was defined as having menstruated in the past three months or no changes in menstrual frequency in the past 12 months; perimenopausal was defined as changes in frequency of menstruation, or between 3–11 months of amenorrhoea; and postmenopausal was defined as amenorrhoea for 12 or more consecutive months [28]. Lifestyle habits assessed included smoking (yes/no), alcohol (yes/no), and the amount of time spent on *moderate* (<150 minutes/≥150 minutes) and/or *vigorous* (<75 minutes/≥75 minutes) intensity physical activity per week, as measured by the Global Physical Activity Questionnaire [36]. The available responses to the question on subjective evaluation of general health status were “poor,” “fair,” “good,” “very good” and “excellent” [37].

#### 2.4.2. Biophysical measurements

Anthropometric measures, including height, weight, waist circumference, and hip circumference were obtained in a standardised manner that has been previously described [28]. Body mass index (BMI) was computed using the formula weight in kg/(height in m)^2^, and was categorised according to the World Health Organization (WHO) revised classification of BMI for Asians: underweight (<18.5 kg/m^2^), normal (18.5–22.9 kg/m^2^), overweight (23.0–27.49 kg/m^2^), and obese (≥27.5 kg/m^2^) [38]. Waist-to-hip ratio was dichotomised as ≤0.80 or >0.80 [39]. Visceral adipose tissue (VAT) in cm^2^ was measured using dual-energy X-ray absorptiometry (DXA) (Hologic Discovery Wi, Apex Software 4.5); daily calibrations were performed by trained operators using a standardised protocol [40]. VAT was categorised into tertiles: low (<88.4 cm^2^), middle (88.5–131 cm^2^), or high (>131 cm^2^) tertile [41].

### 2.5. Statistical methods

All statistical analyses were performed using SPSS (*Version 26*.*0*. Armonk, NY: IBM Corp). Descriptive statistics are presented as count (proportion) for categorical data, mean (SD) for *parametric* continuous data, and median (range) for *non-parametric* continuous data. Differences in categorical variables across language preference/ethnicity groups were assessed using Pearson’s chi-square, while continuous variables were tested with one-way analysis of variance (ANOVA). Bonferroni corrections were applied when multiple pairwise comparisons were made between ELP vs. CLP, ELP vs. Malay, ELP vs. Indian women. Multivariable logistic regression models were constructed to assess independent associations of ethnicity/language with muscle strength and physical performance outcomes, presented as adjusted odds ratios (aORs) with corresponding 95% confidence intervals (CIs). Binary logistic regression models were applied for all outcomes, except for MSI, where multinomial logistic regression was used. Covariates were identified based on significance in crude analyses, evaluated at alpha 0.05. A unique model was applied to each of the outcomes studied (S1 Table).

## 3. Results

### 3.1. Baseline characteristics

1201 (54.8%) women met the inclusion criteria and agreed to participate in the IWHP cohort. Among the 1201 eligible women, we excluded a further 37 women whose ethnicity was outside the three major ethnic groups in Singapore. Of the 1164 women (age: 56.3 ± 6.2 years) included in the analytical cohort, 979 (84.1%) identified as ethnic Chinese, 65 (5.6%) as Malays, and 120 (10.3%) as Indians (Table 1). 32.2% of ethnic Chinese opted for the Chinese version of the validated questionnaire, indicating a preference for Chinese in written communications. Table 1 summarizes the sociodemographic, reproductive health and status, lifestyle habits, and anthropometric/ biophysical measurements of the cohort, stratified by ethnicity and language preference.

**Table 1:** Participant characteristics (n = 1164).

	Overall	Chinese		Malay	Indian	p-value^a^
		(n = 979)				
		*English language preference* (ELP)	*Chinese language preference* (CLP)			
	(n = 1164)			(n = 65)	(n = 120)	
		(n = 664)	(n = 315)			
**Sociodemographic features**						
**Age**	56.3 ± 6.2	56.6 ± 6.3	57.2 ± 6.2	53.5 ± 5.0	53.9 ± 5.4	**<0.001**
**Marital status**						
Married	937 (81.0)	519 (78.6)	266 (84.7)	54 (83.1)	98 (83.1)	0.127
Not married	220 (19.0)	141 (21.4)	48 (15.3)	11 (16.9)	11 (16.9)	
**Highest level of education**						
No formal education or primary	169 (14.7)	27 (4.2)	127 (40.3)	4 (6.2)	11 (9.2)	**<0.001**
Secondary or pre-tertiary	749 (65.2)	441 (68.0)	175 (55.6)	53 (81.5)	80 (67.2)	
Tertiary	230 (20.0)	181 (27.9)	13 (4.1)	8 (12.3)	28 (23.5)	
**Employment status**						
Working	780 (67.4)	467 (70.8)	186 (59.4)	41 (63.1)	86 (72.3)	**0.003**
Not working	377 (32.6)	193 (29.2)	127 (40.6)	24 (36.9)	33 (27.7)	
**Monthly household income (SGD)**						
Less than 3000	291 (28.8)	130 (22.4)	109 (42.9)	20 (32.8)	32 (27.8)	**<0.001**
3000–6999	405 (40.1)	221 (38.1)	104 (40.9)	32 (52.5)	48 (41.7)	
7000 and above	314 (31.1)	229 (39.5)	41 (16.1)	9 (14.8)	35 (30.4)	
**Reproductive Health and Status**						
**Parity**						
Nulliparous	162 (14.0)	126 (19.3)	19 (6.0)	5 (7.7)	12 (10.0)	**<0.001**
Parous	992 (86.0)	528 (80.7)	296 (94.0)	60 (92.3)	108 (90.0)	
**Age of menopause**	49.7 ± 4.4	49.7 ± 4.5	50.5 ± 3.9	47.8 ± 4.1	48.0 ± 4.8	**<0.001**
**Menopausal status**						
Pre-menopausal	148 (12.7)	86 (13.0)	34 (10.8)	8 (12.3)	20 (16.7)	0.058
Peri-menopausal	181 (15.5)	98 (14.8)	42 (13.3)	16 (24.6)	25 (20.8)	
Post-menopausal	835 (71.7)	480 (72.3)	239 (75.9)	41 (63.1)	75 (62.5)	
**Lifestyle Habits**						
**Smoking**						
Yes	23 (2.0)	12 (1.8)	7 (2.2)	1 (1.5)	3 (2.5)	0.933
No	1135 (98.0)	648 (98.2)	307 (97.8)	64 (98.5)	116 (97.5)	
**Alcohol**						
Yes	36 (3.1)	21 (3.2)	13 (4.2)	0 (0.0)	2 (1.7)	0.261
No	1118 (96.9)	637 (96.8)	300 (95.8)	65 (100.0)	116 (98.3)	
**Physical Activity (per week)**						
<150 mins of *moderate* intensity or <75 mins of *vigorous* intensity	445 (38.3)	233 (35.1)	137 (43.5)	37 (56.9)	38 (31.7)	**<0.001**
≥150 mins *moderate* intensity or >75 mins of *vigorous* intensity	718 (61.7)	430 (64.9)	178 (56.5)	28 (43.1)	82 (68.3)	
**Self-rated health status**						
Poor/ Fair	433 (37.7)	227 (34.7)	144 (46.5)	25 (38.5)	37 (31.1)	**0.002**
Good/ Very Good/ Excellent	716 (62.3)	428 (65.3)	166 (53.5)	40 (61.5)	82 (68.9)	
**Anthropometric and biophysical measurements**						
**BMI (kg/m** ^**2**^ **)**						
Underweight (<18.5)	63 (5.5)	47 (7.2)	16 (5.1)	0 (0.0)	0 (0.0)	**<0.001**
Normal (18.5–22.9)	482 (41.9)	302 (46.1)	147 (47.1)	10 (15.4)	23 (19.5)	
Overweight (23–27.49)	393 (34.2)	222 (33.9)	110 (35.3)	17 (26.2)	44 (37.3)	
Obese (≥27.5)	212 (18.4)	84 (12.8)	39 (12.5)	38 (58.5)	51 (43.2)	
**Waist circumference**						
<80cm	531 (45.9)	359 (54.5)	146 (46.6)	9 (13.8)	17 (14.3)	**<0.001**
≥80cm	625 (54.1)	300 (45.5)	167 (53.4)	56 (86.2)	102 (85.7)	
**Waist-to-hip ratio**						
≤0.80	282 (24.5)	183 (28.0)	56 (17.9)	18 (27.7)	25 (21.0)	**0.005**
>0.80	869 (75.5)	471 (72.0)	257 (82.1)	47 (72.3)	94 (79.0)	
**Visceral Adipose Tissue (cm** ^**2**^ **)**						
Lowest tertile	385 (33.3)	254 (38.5)	111 (35.6)	7 (10.8)	14 (11.8)	**<0.001**
Middle tertile	389 (33.7)	212 (32.2)	107 (34.3)	22 (33.8)	47 (39.5)	
Highest tertile	381 (33.0)	193 (29.3)	94 (30.1)	36 (55.4)	58 (48.7)	

^a^ Significance was established using Person’s Chi-square test for categorical variables and one-way ANOVA for continuous variables, comparing the outcomes of ethnic Chinese with English language preference vs. ethnic Chinese with Chinese language preference vs. Malay vs. Indians.

Data is presented as count (proportion) for categorical data and mean ± standard deviation for continuous data.

### 3.2. Comparison of participant-related baseline characteristics

#### 3.2.1. Sociodemographic features

Chinese women (ELP and CLP group) were significantly older and more often post-menopausal compared to Malay and Indian women (Table 1). Among Chinese women, those with a Chinese language reading preference (CLP) were more often parous (94.0% vs 80.7%), less likely to have completed tertiary education (4.1% vs 27.9%), less likely to be employed (59.4% vs 70.8%), and less likely to have a monthly household income of SGD7000 or above (16.1% vs 39.5%) compared to those with an English-language preference (ELP). The CLP group also had engaged in less physical activity (≥150 min a week) (56.5% vs 64.9%) and were more inclined to report their health as poor/ fair (46.5% vs 34.7%) compared to the ELP group.

Compared to ELP women, Malay women were more likely to be parous (92.3% vs 80.7%), less likely to have received tertiary education (12.3% vs 27.9%) and less likely to report a monthly household income of ≥SGD7000 (14.8% vs 39.5%). Malay participants were also more likely to report lower levels of physical activity compared to the ELP group (43.1% vs 64.9%).

Indian women, compared to ELP subjects, were more often parous (90.0% vs 80.7%) but did not differ significantly in other sociodemographic characteristics.

No significant differences were observed in the proportion of participants who smoked or drank alcohol across the four ethnicity/language groups.

#### 3.2.2. Anthropometric and biophysical measurements

No significant differences in BMI, or VAT were observed between CLP and ELP Chinese. In marked contrast, levels of adiposity were higher among women of Malay and Indian ethnicity compared to Chinese women, irrespective of language preference. Malay and Indian women were, respectively, more likely to be obese (58.5%, 43.2% **vs** 12.8%, 12.5%), more likely to have a waist circumference ≥80cm (86.2%, 85.7% **vs** 45.5%, 53.4%), and more likely to be in the highest tertile of VAT (55.4%, 48.7% **vs** 29.3%, 30.1%) compared to the ELP and CLP groups.

#### 3.2.3. Objectively assessed physical performance

Objectively assessed physical performance outcomes are presented in Table 2. We observed marked differences in muscle strength and objectively measured physical performance between the two Chinese groups. Compared to their ELP counterparts, a significantly higher proportion of CLP participants had weak hand grip strength (28.5% vs 16.7%), poor repeated chair stand test (57.0% vs 39.8%), and consequently poor combined muscle strength index (19.7% vs 8.5%) (Table 2). The balance tests of CLP women were also significantly poorer: with short tandem stand (8.6% vs 5.0%) and one-leg stand (29.2% vs 21.7%). A significantly greater proportion of CLP women had slow gait speeds, both in the usual (20.1% vs 12.6%) and narrow walks (24.3% vs 16.2%) compared to Chinese women with ELP.

**Table 2:** Objectively assessed physical performance.

	Overall	Chinese		Malay	Indian	p-value^a^
		(n = 979)				
		*English language preference*	*Chinese language preference*			
	(n = 1164)			(n = 65)	(n = 120)	
		(ELP)	(CLP)			
		(n = 664)	(n = 315)			
**Handgrip Strength (HGS)**						
Handgrip Strength (HGS) <18 kg	242 (21.2)	109 (16.7)	88 (28.5)	16 (24.6)	29 (24.8)	**<0.001**
Handgrip Strength (HGS) ≥18 kg	900 (78.8)	542 (83.3)	221 (71.5)	49 (75.4)	88 (75.2)	
**Repeated Chair Stand (RCS)**						
Repeated Chair Stand (RCS) ≥ 12 s	511 (45.7)	251 (39.8)	176 (57.0)	28 (45.2)	56 (48.7)	**<0.001**
Repeated Chair Stand (RCS) < 12 s	606 (54.3)	380 (60.2)	133 (43.0)	34 (54.8)	59 (51.3)	
**Muscle Strength Index (MSI)**						
Poor (HGS <18kg AND RCS ≥12 s)	143 (13.0)	53 (8.5)	60 (19.7)	10 (16.1)	20 (17.9)	**<0.001**
Intermediate (HGS <18kg OR RCS ≥12 s)	443 (40.3)	235 (37.9)	140 (45.9)	23 (37.1)	45 (40.2)	
Normal (HGS ≥18 kg AND RCS <12 s)	513 (46.7)	332 (53.5)	105 (34.4)	29 (46.8)	47 (42.0)	
**Balance Tests (seconds)**						
Semi-tandem stand < 30 s	28 (2.4)	14 (2.1)	2 (3.1)	2 (1.7)	10 (3.2)	0.695
Semi-tandem stand ≥ 30 s	1136 (97.6)	650 (97.9)	305 (96.8)	63 (96.9)	118 (98.3)	
Tandem stand < 30 s	68 (5.8)	33 (5.0)	27 (8.6)	4 (6.2)	4 (3.3)	0.087
Tandem stand ≥ 30 s	1096 (94.2)	631 (95.0)	288 (91.4)	61 (93.8)	116 (96.7)	
One leg stand < 30 s	288 (24.7)	144 (21.7)	92 (29.2)	13 (20.0)	39 (32.5)	**0.010**
One leg stand ≥ 30 s	876 (75.3)	520 (78.3)	223 (70.8)	52 (80.0)	81 (67.5)	
**Gait speed**						
Usual walk < 1m/s	181 (15.6)	83 (12.6)	63 (20.1)	10 (15.4)	25 (20.8)	**0.008**
Usual walk ≥ 1m/s	976 (84.4)	576 (87.4)	250 (79.9)	55 (84.6)	95 (79.2)	
Narrow walk < 1m/s	223 (19.3)	107 (16.2)	76 (24.3)	8 (12.3)	32 (26.7)	**0.002**
Narrow walk ≥ 1m/s	934 (80.7)	552 (83.8)	237 (75.7)	57 (87.7)	88 (73.3)	

^a^ Significance was established using Person’s Chi-square test for categorical variables, comparing the outcomes of ethnic Chinese with English language preference vs. ethnic Chinese with Chinese language preference vs. Malay vs. Indians. Data is presented as counts (proportion) for categorical data. The percentage of data not available ranged from 3.1% to 8.5%.

No significant differences were observed in the proportions of Malay and Indian women in the handgrip strength or repeated chair stand tests compared to either the ELP or CLP group.

Indian women were about twice as likely to have a ‘poor’ outcome on the muscle strength index compared to ethnic Chinese with ELP (17.9% **vs** 8.5%). Indian women were less likely to balance for 30 seconds on one leg compared to women with ELP (32.5% vs 21.7%). Indian women were significantly more likely to have a slow gait speed, both in the usual (20.8% vs 12.6%) and narrow walk test (26.7% vs 16.2%) compared to Chinese women with ELP.

### 3.3. Adjusted analyses for associations of ethnicity/ language preference and physical performance

Table 3 summarises the results of the multivariable logistic regression, depicting the associations between objectively measured muscle strength and physical performance indices across the four groups. CLP women were about 1.5 to 2.5 times more likely to have low muscle strength compared to the ELP group. The CLP group were more likely to have weak hand grip strength (adjusted OR (aOR) 1.56, 95% CI: 1.07–2.27) and a slow repeated chair stand test (aOR 1.55, 95% CI: 1.12–2.13) compared to the ELP group (Table 3). Using the muscle strength index (MSI) as a composite index to combine upper and lower body strength, CLP women were 2.44 times (95% CI: 1.47–4.04) more likely to have poor performance versus ELP women. Chinese language preference was also associated with poor balance, as demonstrated by a low tandem stand duration (aOR 2.00, 95% CI: 1.16–3.47) and impaired functional mobility with a slow gait speed (aOR 1.62, 95% CI: 1.06–2.47). Adjusted associations between reading language preference and gait speed (narrow walk) and one-leg stand performance were not significant.

**Table 3:** Adjusted odds ratio (aOR) and 95% confidence intervals (CI) for associations of reading language preference and ethnicity with objective measures of physical performance, based on multivariable logistic regression models with ethnic Chinese with English language preference (ELP) as the reference group.

	Ethnic Chinese with Chinese-Language Preference (CLP)	Malays	Indians
	aOR (95% CI)	aOR (95% CI)	aOR (95% CI)
**Maximum handgrip strength < 18kg** ^1^	1.56 (1.07–2.27)*	1.81 (1.12–2.93)*	1.84 (0.99–3.40)
**Repeated Chair Stand > 12 sec** ^2^	1.55 (1.12–2.13)*	1.15 (0.66–2.03)	1.38 (0.90–2.14)
**Muscle Strength Index** ^**3**^			
Poor	2.44 (1.47–4.04)*	3.07 (1.36–6.96)*	3.31 (1.75–6.28)**
Intermediate	1.48 (1.05–2.08)*	1.13 (0.63–2.04)	1.47 (0.93–2.33)
**Balance tests**			
Semi-tandem stand < 30 secs^4^	—	—	—
Tandem stand < 30 secs^5^	2.00 (1.16–3.47)*	1.98 (0.66–5.96)	0.96 (0.33–2.84)
One leg stand < 30 secs^6^	1.17 (0.79–1.74)	0.94 (0.46–1.93)	2.12 (1.28–3.52)*
**Gait speed**			
Usual walk < 1m/s^7^	1.62 (1.06–2.47)*	1.29 (0.61–2.77)	1.88 (1.09–3.25)*
Narrow walk < 1m/s^8^	1.19 (0.78–1.77)	0.72 (0.32–1.62)	1.91 (1.15–3.17)*

*p-value <0.05

**p-value <0.001

Compared to Chinese women with ELP, Malay women had a higher odds of a weak hand grip strength (aOR 1.81, 95% CI: 1.12–2.93). While they were not significantly more likely to show a poor repeated chair stand on adjusted analyses, a poor MSI composite index was three times more likely among Malay women (aOR 3.07, 95% CI: 1.36–6.96) than among Chinese women who preferred the English version of the questionnaire. Multivariable analyses did not reveal any significant associations of language preference/ethnicity on balance tests or gait speed.

Similarly, Indian women were 3.31 times (95% CI: 1.75–6.28) more likely to have a poor performance on the MSI compared to ELP women. MSI revealed significant differences in combined body strength that were not demonstrated by individual indices (handgrip strength or repeated chair stand test). Indian women were also twice as likely to have a poor performance on the one-leg stand test (aOR 2.21, 95% CI: 1.28–3.52) and a slow gait speed on both a usual path (aOR 1.88, 95% CI: 1.09–3.25) and narrow path (aOR 1.91, 95% CI: 1.15–3.17) compared to ELP women.

## 4. Discussion

We observed that among mid-life women in Singapore, ethnicity and reading language preference were associated with muscle strength and physical performance. Differences in muscle strength and physical performance from the majority English-reading language preference (ELP) group were the largest and most consistent in the Chinese-reading language preference (CLP) group, followed by Indian and Malay women. Even after adjustments, the CLP group demonstrated inferior outcomes across almost all indicators of physical performance, including poor upper and lower extremity strength, poor balance, and slow gait speed compared to their ELP counterparts. These associations were independent of age and other important covariates.

It was surprising to observe substantial differences in muscle strength and function according to reading language preference within a majority ethnic group, in a developed city-state with an advanced healthcare infrastructure [42]. Disparities in muscle strength and physical performance have also been identified in Chinese-American, but not Japanese-American, mid-life women. The mean decile muscle performance scores of Chinese-Americans were 2.1 points lower than for Non-Hispanic White women, with differences by ethnicity that would have been even larger if not for the lower BMI in the Chinese [12].

In our study, the reasons for the CLP association with muscle strength and performance, but not with BMI, or VAT are unclear. One possible mediator may be lower social economic status (SES), as CLP women were less likely to have completed tertiary education, were more often parous, were less likely to be employed and had a lower income. We adjusted for these differences, but results remained similar. This may reflect the difficulty in measuring the social determinants of health. Lower SES has been correlated with unfavourable trajectories of physical performance, frailty, and disability [43]. Language preference may reflect a greater degree of attachment and endorsement of cultural values such as familism, and self-sacrifice [44]. Duties as a wife, mother, and burdensome household responsibilities could perhaps influence the allocation of time away from selfcare, aerobic and other muscle strengthening exercises [44, 45].

Another possible factor may be the inability to access community health information services, wherein Chinese respondents in a previous study who were not predominantly speakers of English were twice as likely to report feeling treated “worse” or “much worse” than those who spoke English when seeking hospital services, compared to English speakers [46]. Only 11% and 6% of Malay and Indian speakers indicated likewise, respectively [46]. These negative experiences while seeking health information reinforces the need for health communication to serve the needs of non-English speaking populations. Methods of health information dissemination, patient education materials for instance, should be re-evaluated and created for the target audience. Factors such as the target audience’s demographics, language preference and preferred mode of communication channels should be considered to promote an equitable access to health [47].

Malay women also exhibited lower SES, with higher proportion being parous, lower proportion with tertiary education and a lower household income compared to the ELP Chinese. Besides these factors, our model may have failed to account for other dimensions of SES which may have resulted in the association of a three-fold poorer muscle strength index compared to the ELP group. Indian women, who did not differ significantly in other sociodemographic characteristics from the ELP Chinese group had a muscle strength index that was more than 3-fold poorer. In another study, Malay and Indian participants had poorer hand grip strength compared to Chinese participants after adjusting for other sociodemographic correlates [48, 49].

Many women in the IWHP cohort, with a mean age of 56 years at the time of enrolment in 2014, commenced their formal education as English was increasingly adopted in Singapore as the language of instruction [50]. Whilst many prefer to use English both officially and at home, a significant proportion of midlife women retained Chinese as their preferred reading language, providing a unique opportunity to identify health outcomes associated with language preference in Singapore [51].

In the longer term, poor muscle strength and performance may presage increasing frailty, higher morbidity, and mortality. Indeed, systematic reviews have consistently identified muscle strength as an independent risk factor for all-cause mortality [4, 9]. If confirmed in longitudinal studies, our findings may suggest the need to tailor health interventions to improve muscle function according to language preference. Particularly for the CLP group, and those of Malay and Indian ethnicities, a greater emphasis might need to be placed on progressive resistance or weight-based training and optimising nutrition to address their disparities in muscle strength and physical performance [52].

Among limitations of our study, our restriction to health-seeking subjects may not be generalisable to other populations. Nevertheless, our data may be relevant to health inequalities in Chinese populations in North America [53–56], United Kingdom [57] and Australia [58] in whom first- or even second-generation Chinese immigrants may still prefer their native language. Language preferences for Malay and Indian ethnicities were not elicited in our study, because of the small number of participants in these groups. The unequal sample sizes between the three ethnicities may have contributed to a reduced power and increased the probability of Type I error [59]. Notably, our study relied exclusively on the self-reported ethnicity of participants. For participants of mixed ancestry, classification according to the ethnic group of the father may over-simplify the complexity of ethnic differences [60]. Finally, despite our attempts to adjust for other predictive variables, other unknown or unmeasured factors could have influenced our observations. Future studies should explore these sociocultural, behavioural, and lifestyle factors to elucidate the longitudinal effects of intra- and inter-ethnic differences on muscle strength and physical performance and consequent health and disease patterns.

Our study also has several important strengths. To the best of our knowledge, no previous study has examined reading language preference and objectively measured physical performance within a single ethnic group. Disentangling language preference from ethnicity enhances our understanding of *subtle* sociocultural differences that may affect health outcomes. By analysing the individual components of objectively measured physical performance, we were able to identify strong associations between language preference and muscle strength and performance in Chinese women.

## 5. Conclusion

Chinese language reading preference was associated with poorer muscle strength and physical performance, suggesting a possible need to include language preference in health risk assessments for Chinese women. Future studies of health disparities should disaggregate language subgroups within ethnicities. Public health messages in Singapore might need to emphasise the importance of physical activity to increase muscle strength, balance, and performance in all ethnicities, while tailoring those messages to women preferring Chinese as their language of communication. In the backdrop of an uptrend in international migration, our findings may potentially be extrapolated to subgroups of the population who are not proficient in the *lingua franca*.

## Supporting information

S1 Table List of covariates included in multivariable logistic regression models. (DOCX)
