# Supplementary material for: Associations of reading language preference with muscle strength and physical performance: Findings from the Integrated Women’s Health Programme (IWHP)
Source: PLoS One. 2023 Apr 10;18(4):e0284281. doi: 10.1371/journal.pone.0284281 (PMC10085028; doi:10.1371/journal.pone.0284281)
Supplement: S1 Table — (DOCX) [file pone.0284281.s001.docx]

**Supplementary Table 1:** List of covariates included in multivariable logistic regression models

| **Outcome** | Age | Marital Status | Highest Education Level | Menopausal Status | BMI |
| --- | --- | --- | --- | --- | --- |
| Hand Grip Strength | ✓ |  | ✓ |  |  |
| Repeated Chair Stand Test | ✓ |  | ✓ | ✓ | ✓ |
| Muscle Strength Index | ✓ |  | ✓ | ✓ |  |
| Semi-Tandem Stand |  |  |  |  |  |
| Tandem Stand | ✓ | ✓ |  | ✓ |  |
| One-leg Stand | ✓ |  | ✓ | ✓ | ✓ |
| Gait speed (usual walk) | ✓ |  | ✓ |  |  |
| Gait speed (narrow walk) | ✓ |  | ✓ | ✓ |  |
